# Vascular Endothelial Growth Factor in Tear Samples of Patients with Systemic Sclerosis

**DOI:** 10.1155/2015/573681

**Published:** 2015-08-03

**Authors:** Anikó Rentka, Jolán Hársfalvi, András Berta, Krisztina Köröskényi, Zoltán Szekanecz, Gabriella Szücs, Peter Szodoray, Ádám Kemény-Beke

**Affiliations:** ^1^Department of Ophthalmology, University of Debrecen, Nagyerdei Körút 98, Debrecen 4012, Hungary; ^2^Department of Biophysics and Radiation Biology, Semmelweis University, Tűzoltó Utca 37-47, Budapest 1094, Hungary; ^3^Institute of Biochemistry and Molecular Biology, Signaling and Apoptosis Research Group, Hungarian Academy of Sciences, Research Center of Molecular Medicine, Nagyerdei Körút 98, Debrecen 4012, Hungary; ^4^Department of Rheumatology, Institute of Medicine, Faculty of Medicine, University of Debrecen, Nagyerdei Körút 98, Debrecen 4012, Hungary; ^5^Institute of Immunology, Rikshospitalet, Oslo University Hospital, Sognsvannsveien 20, 0372 Oslo, Norway

## Abstract

*Background*. Systemic sclerosis is an autoimmune disease, characterized by widespread small vessel vasculopathy, immune dysregulation with production of autoantibodies, and progressive fibrosis. Changes in levels of proangiogenic cytokines had already been determined largely in serum. Our aim was to assess the levels of VEGF in human tears of patients with SSC. *Patients and methods*. Forty-three patients (40 female and 3 men, mean (SD) age 61 (48–74) years) with SSc and 27 healthy controls were enrolled in this study. Basal tear sample collection and tear velocity investigations were carried out followed by an ophthalmological examination. Total protein concentrations and VEGF levels were determined in tear samples. *Results*. The average collected tear fluid volume developed 10.4 *μ*L (1.6–31.2) in patients and 15.63 *μ*L (3.68–34.5) in control subjects. The average total protein level was 6.9 *μ*g/*μ*L (1.8–12.3) in tears of patients and control tears contained an average of 4.132 *μ*g/*μ*L (0.1–14.1) protein. In patients with SSc the average concentration of VEGF was 4.9 pg/*μ*L (3.5–8.1) and 6.15 pg/*μ*L (3.84–12.3) in healthy samples. *Conclusions*. Total protein production was increased because of the smaller tear volume. Decreased VEGF in tear of SSc patients can be explained also by the decreased tear secretion of patients.

## 1. Introduction

Systemic sclerosis (SSc or scleroderma) is a chronic connective tissue disorder which affects mostly the skin and multiple internal organs, including the heart, lung, kidneys, and gastrointestinal tract. SSc is a systemic autoimmune disease characterized by widespread small vessel vasculopathy, immune dysregulation with production of autoantibodies, and progressive fibrosis [1].

There are only few reports available on the ophthalmological complications during the course of systemic sclerosis. Changes in the organ of vision are thought to be the consequence of systemic complications of scleroderma or adverse effects of immunosuppressive treatment applied. Ocular symptoms may occur at any stage of the disease and may involve numerous ocular tissues. Their course can be clinically latent or very intensive. The most common clinical manifestations of soft tissue fibrosis and inflammation in these patients include increased tonus and telangiectasia of eyelid skin. The most commonly reported lesions are periorbital edema, palpebral ectropion, and ciliary madarosis [2]. In our study the most frequent ocular manifestation of SSc was dry eye syndrome (DES).

DES is a major healthcare problem since it affects the patients' quality of life. DES was recently redefined as a multifactorial disease of the tears and ocular surface that results in symptoms of discomfort, visual disturbance, and tear film instability and after all damage to the ocular surface [3]. Increased osmolality of the tear film [4] and inflammation of the ocular surface [5] are the two major characteristic points in DES. Increased levels of several inflammatory cytokines are the most important laboratory findings [6]. Accordingly, tear cytokine levels are already considered as potential markers of inflammation in DES.

Although the exact origin of SSc is unknown, some predisposing factors—like environmental and infectious agents, tissue injury, and hypoxia or oxidative stress on a susceptible genetic background—may play a role in the disease development [1, 7]. One of the earliest clinical manifestations in the pathophysiology of SSc is vascular injury that can be caused by the imperfect angiogenesis due to impaired production of proangiogenic factors. Vascular endothelial growth factor (VEGF) is one of the most important proangiogenic factors which play a key role in the formation of new blood vessels [8]. VEGF takes part in various steps of angiogenesis, including initial vasodilation, endothelial cell permeability, perivascular matrix remodeling, and induction of proliferation and migration of endothelial cells [9].

Although tear analysis is of increasing interest in ophthalmology, yet no studies have investigated tears of SSc patients, possibly because of its technical challenge due to the small sample volumes available [10].

The aim of this study was to demonstrate a significant difference of VEGF levels in human tears of SSc patients compared to healthy controls. Since tear dynamically reflects the factors present at the ocular surface, this is an ideal sample to investigate; furthermore, it has the potential for earlier diagnosis of SSc with a noninvasive method of collecting samples.

## 2. Materials and Methods

### 2.1. Patients and Healthy Controls

Forty-three patients with SSc and 27 healthy controls were included in our study. Patients were enrolled from the outpatient clinic at the Department of Rheumatology, Department of Ophthalmology (40 female and 3 men), mean (SD) age 61 (48–74) years. SSc was diagnosed based on the corresponding international criteria [11, 12].

They went through ophthalmological examination and basal tear sample collection at the Department of Ophthalmology during a 12-month period. DES was diagnosed in 32 of 43 SSc patients. All patients have undergone a broad immunoserological screening, including ANA, anti-SSA, anti-SSB, corresponding ophthalmological tests, and salivary measurement, which excluded the presence of coexisting secondary Sjogren's syndrome.

We enrolled 27 volunteers (21 female and 6 men) as healthy controls who had no history of any autoimmune or ocular disorder. Patients did not take immunosuppressive medications at the time of the tear sampling.

Written informed consent was obtained from all patients and controls. Study protocol was approved by the local bioethics committee and followed the tenets of the declaration of Helsinki.

### 2.2. Tear Sample Collection

Open eye tears were gently collected from the inferior temporal meniscus of both eyes using a capillary micropipette (Haematokrit-Kapillaren, Na-Heparin 3.0 IU/kapillare, 75 mm/60 *μ*L, Hischmann Laborgerate), minimizing irritation of the ocular surface or lid margin as much as possible. All samples were collected between 11 am and 16 pm by the same physician. Tear secretion velocity was counted by dividing the volume of collected sample by time of secretion. Volume was calculated from the lengths of the fluid column in the capillary tube, measured with a vernier caliper, and from the known diameter of the tube. Time of tear collection was measured with stopwatch. Tears were transferred into low binding capacity Eppendorf tubes by washing out the capillaries five times with PBS-T buffer equal to the volume of tears. Fivefold diluted samples were stored at −80°C until assessment.

### 2.3. Tear Sample Preparation and Quantification

First, as a point of reference for VEGF, total protein concentrations were determined in tear samples with the BCA Microplate method (Sigma-Aldrich, St. Louis, MO, USA) according to the manufacturer's instructions. Human albumin was used as standard.

For the quantitative determination of VEGF in tear fluid we used a human VEGF immunoassay kit by Quantikine (R&D Systems, Minneapolis, MN, USA) according to the manufacturer's instructions. This assay employs the quantitative sandwich enzyme immunoassay technique.

## 3. Statistical Analyses

Prism 5 statistical software (GraphPad Software Inc.) was used for statistical analyses. Comparison of values was carried out by Mann-Whitney *U* test. All values are shown as the mean ± SD. *P* values less than 0.05 were considered statistically significant.

## 4. Results

The average tear secretion velocity in patients was 4.53 *μ*L/min, with a median of 3.8 *μ*L/min (1.5–25.6).

Duration of tear sample collection from patients changed between 20 and 313 seconds until the minimally required 5 *μ*L volume was reached.

The average collected tear fluid volume developed 10.4 *μ*L (1.6–31.2) in case of patients and 15.63 *μ*L (3.68–34.5) in case of controls.

**Figure 1:**
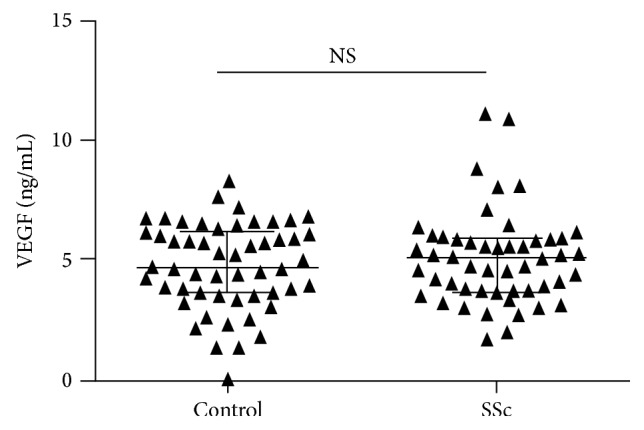
VEGF concentrations (ng/mL) in tears of healthy controls (left) and SSc patients (right).

In tear samples of patients with SSc the average total protein level was 6.9 *μ*g/*μ*L (1.8–12.3) and the average concentration of VEGF was 4.9 pg/*μ*L (3.5–8.1) in case of basal tear secretion (Figure 1).

**Figure 2:**
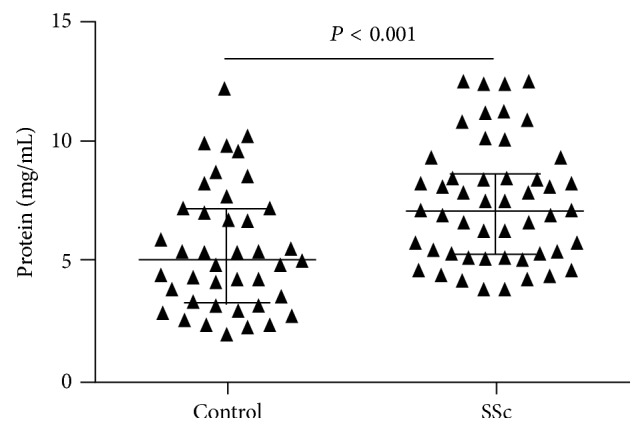
Total protein concentrations (mg/mL) in tears of healthy controls (left) and SSc patients (right).

Control tears contained an average of 4.132 *μ*g/*μ*L (0.1–14.1) protein and 6.15 pg/*μ*L (3.84–12.3) VEGF (Figure 2).

## 5. Discussion

Systemic sclerosis is an autoimmune disease affecting the connective tissue and characterized by a wide spectrum of microvascular and immunological abnormalities, leading to progressive fibrosis of the skin and other visceral organs, such as lungs, gastrointestinal tract, heart, and kidneys [13]. Many ocular manifestations of SSc have been described including conjunctival telangiectasia, DES, and filamentous keratitis [14].

DES in SSc is believed to be caused by fibrosis-related impairment of lacrimal gland secretion, namely, the water portion of the tear film. Furthermore, the lipid layer disorder is caused by chronic blepharitis and meibomian gland dysfunction (MGD), and also the increased evaporation of tears from the ocular surface is the consequence of the restricted eyelid mobility and the consecutive reduced blinking [15].

Tear plays an essential role in maintaining homeostasis of the ocular surface; therefore changes in the delicate equilibrium of its cytokine composition may lead to various pathophysiological conditions.

The influence of VEGF in ophthalmic diseases is profound. It has been implicated in a large number of retinal diseases and conditions like age-related macular degeneration and diabetic retinopathy, retinopathy of prematurity, sickle cell retinopathy, and retinal vascular occlusion. VEGF has secondary influence in neovascular glaucoma [16] and inherited retinal dystrophies [17]. Since it has been discovered in the 1980s [18] VEGF has raised interest because of its central role in angiogenesis in a number of physiologic and pathologic processes, such as vascular development, wound healing, the female reproductive cycle, cancers, myocardial ischemia, rheumatoid arthritis, and other autoimmune diseases. VEGF is a component of normal tear fluid. Vesaluoma determined VEGF concentrations in healthy tears. The median VEGF concentration was 5 pg/*μ*L (4–11) which corresponds with our results, as control tears contained an average of 6.15 pg/*μ*L (3.84–12.3) VEGF. Vesaluoma and co-workers calculated the average tear fluid secretion in healthy controls, which was 8.1 *μ*L/min (0.7–20.8), using the same tear collecting method as we used in our study. Results show that SSc patients have significantly decreased tear secretion that could be explained by DES, which is a probable sequel of the disease or of the side effects of the therapeutic drugs [19].

Tear secretion velocity was lower by 67% in SSc patients than in healthy controls. The difference was significant (*P* < 0.01). The reason for this sign could be explained by the pathophysiology of the disease, namely, fibrotic processes.

Total protein values in SSc patients were higher by 42% than in healthy controls. It may indicate that total protein production—or simply the protein concentration, since SSc patients have a decreased tear secretion velocity—is only increased because of the smaller tear volume. Though VEGF in tear of SSc patients was decreased by 20% and did not change after stimulation, it can be explained also by the decreased tear secretion of patients.

Although the average VEGF level in tear collected without stimulation was higher than that in tear collected with stimulation in SSc patients, the difference was not significant. The phenomenon why the VEGF levels are not higher in SSc patients than in the healthy group needs further investigations.

## Abbreviations

SSc: Systemic sclerosis
DES: Dry eye syndrome
MGD: Meibomian gland dysfunction
VEGF: Vascular endothelial growth factor
MMP: Matrix metalloproteinase
MCP: Monocyte chemoattractant protein
IL: Interleukin
IP: Interferon gamma-induced protein
TNF: Tumor necrosis factor
FasL: Fas ligand.
